# Effects of dietary supplementation with two alternatives to antibiotics on intestinal microbiota of preweaned calves challenged with *Escherichia coli* K99

**DOI:** 10.1038/s41598-017-05376-z

**Published:** 2017-07-14

**Authors:** Yanliang Bi, Chuntao Yang, Qiyu Diao, Yan Tu

**Affiliations:** Feed Research Institute, Chinese Academy of Agricultural Sciences, Key Laboratory of Feed Biotechnology of the Ministry of Agriculture, 100081 Beijing, China

## Abstract

The aim of this study was to investigate the effects of dietary supplementation with two alternatives to antibiotics (*Candida tropicalis* and mulberry leaf flavonoids) on intestinal microbiota of preweaned calves challenged with *Escherichia coli* K99. Sixty Holstein calves were randomly assigned to 5 treatments: fed a basal diet (N-CON); fed a basal diet and challenged with *E.coli* K99 (P-CON); fed a basal diet supplemented with *C.tropicalis* (CT), mulberry leaf flavonoids (MLF), and the combination of the two additives (CM), respectively, and challenged with *E.coli* K99. The MLF and CM groups had significantly higher average daily grain and feed efficiency, and significantly lower fecal scores compared with the P-CON group after *E. coli* K99 challenge. The supplementation groups increased the relative abundance, at the phylum level, of *Bacteroidetes* and *Proteobacteria*, whereas at the genus level, they increased the relative abundance of *Prevotella*, *Lactobacillus*, and *Enterococcus*. Quantitative PCR revealed that the CT, MLF, and CM groups had significantly lower copy numbers of *E.coli* K99 compared with the P-CON group. The CT, MLF, and CM treatments reduce days of diarrhea, improve intestinal health, and beneficially manipulate the intestinal microbiota in preweaned calves.

## Introduction

Neonatal calf diarrhea is a serious health and welfare problem on dairy farms, with the resulting high mortality and morbidity contributing to considerable economic losses worldwide in the cattle industry^1–3^. Enterotoxigenic *Escherichia coli* expressing K99 fimbriae and heat-stable type Ia (STa) toxin is one of the major pathogens associated with neonatal calf diarrhea^4–6^. The K99 fimbrial adhesins promote attachment and colonization of bacterial cells to the surface of epithelial cells of the small intestines, while the STa toxin damages the epithelial cells and disrupts fluid homeostasis, resulting in fluid and electrolyte hypersecretion that leads to watery diarrhea, dehydration, and acidosis in neonatal calves^7–9^.

Although antibiotics are given therapeutically after scours is observed, concerns have been raised regarding microbial resistance to antibiotics and increasing passage of laws banning the use of antibiotics in livestock production throughout the world. Therefore, alternatives to antibiotics for prevention and treatment of neonatal calf diarrhea are urgently needed to maintain the health of livestock. Recently recognized alternatives include yeasts and flavonoid-containing plant extracts, which are showing beneficial effects on animal intestinal health in an increasing number of studies^10–13^.

*Candida tropicalis*, a yeast of the *Candida* genus, is considered an important inhabitant of the healthy animal gut, and it can commonly be isolated from the gastrointestinal tracts of humans^14^, bovines^15^, birds^16^, and fish^17^. Previous ruminal fermentation studies demonstrated that *C. tropicalis* stimulated total and cellulolytic microbial populations, increased gas production, and activated *in vitro* ruminal fermentation, indicating its excellent potential for use as a feed additive in ruminants^15, 18, 19^. Long *et al*. reported *C. tropicalis* stimulated lactate uptake by *Selenomonas ruminantium* and increased the production of acetate and propionate and the ratio of propionate to acetate^20^. *C. tropicalis* isolated from some fish gastrointestinal tracts increased phytase and tannase production, as well as crude protein, lipid, and mineral contents, and reduced the antinutritional factors in different plant feedstuff^17, 21^.

Plant-derived flavonoids, such as those extracted from mulberry (*Morus alba*) leaves, have also shown health-promoting properties due to the alteration of the expression and activity of key enzymes in lipid and carbohydrate metabolism^22, 23^, induction of protective effects against hydroxyl and superoxide radical damage^24^, antimicrobial activity^25, 26^, antiparasitic activity^27^, and antioxidant activity^24, 28^, among others. Previous studies have shown that flavonoids isolated from the leaves of many different plants exhibit antimicrobial activity against Gram-negative and Gram-positive bacteria and fungi in *in vitro* antimicrobial assays^29–34^. Phytochemical investigations have shown that lipophilic flavonoids exert their antimicrobial activities through their ability to penetrate biological membranes^35^. Omosa *et al*.^26^ demonstrated that the antibacterial activity depended on the relative positions of the hydroxyl and methoxy groups on the flavone skeleton, and that a methoxy group at the C-3 position in the flavone skeleton was associated with good activity against *E. coli*. Several studies have demonstrated that probiotic yeasts might have inhibitory activity against specific pathogens^36–38^, but very few experimental and clinical trials have examined *C. tropicalis* as a possible probiotic. A considerable number of studies have shown that flavonoids isolated from different plants demonstrate antimicrobial activity, but most of these have been *in vitro* investigations. The aim of the present study was therefore to examine the effects of dietary supplementation with *C. tropicalis* and mulberry leaf flavonoids, singly or in combination, on the intestinal bacterial community composition in preweaned calves challenged with *E. coli* K99.

## Results

### Growth performance of calves

In order to determine the effects of *C. tropicalis*, mulberry leaf flavonoids, and their combination on growth performance of calves before and after *E. coli* K99 challenge, the average daily grain (ADG), dry matter intake (DMI), and feed efficiency were analyzed (Table 1). Before *E. coli* K99 challenge, the ADG of N-CON, P-CON, CT, MLF, and CM groups were 0.60, 0.59, 0.62, 0.69, and 0.70 kg/d, respectively. The ADG and DMI had no significant difference among groups, but the feed efficiency of calves in the MLF group was significantly higher than that in the control groups. After *E. coli* K99 challenge, the ADG of N-CON, P-CON, CT, MLF, and CM groups were 0.89, 0.56, 0.62, 0.85, and 0.92 kg/d, respectively. The ADG and feed efficiency of calves in the MLF and CM groups were significantly higher than that in the P-CON and CT groups, but had no significant difference compared with that in the N-CON group. The DMI had no significant difference among groups.

**Table 1:** Effects of *Candida tropicalis*, mulberry leaf flavonoids, and their combination on growth performance of calves.

Item	Treatments					*P*
	N-CON	P-CON	CT	MLF	CM	
28–56 d (before challenge)						
Average daily gain, kg/d	0.60 ± 0.11	0.59 ± 0.12	0.62 ± 0.11	0.69 ± 0.08	0.70 ± 0.11	0.29
Dry matter intake, kg/d	1.17 ± 0.10	1.16 ± 0.11	1.13 ± 0.09	1.14 ± 0.09	1.23 ± 0.12	0.85
Feed efficiency^1^, %	51.28 ± 3.67^b^	51.30 ± 3.30^b^	54.87 ± 4.51^ab^	60.53 ± 3.46^a^	56.91 ± 4.63^ab^	0.02
57–63 d (after challenge)						
Average daily gain, kg/d	0.89 ± 0.11^a^	0.56 ± 0.12^b^	0.62 ± 0.14^b^	0.85 ± 0.13^a^	0.92 ± 0.11^a^	<0.0001
Dry matter intake, kg/d	1.43 ± 0.12	1.38 ± 0.16	1.43 ± 0.10	1.40 ± 0.18	1.47 ± 0.14	0.65
Feed efficiency%	62.40 ± 6.94^a^	40.95 ± 7.35^b^	43.29 ± 8.76^b^	61.34 ± 7.52^a^	62.51 ± 7.30^a^	<0.0001

^1^Feed efficiency = (Average daily gain (kg/d)/Dry matter intake (kg/d)) × 100%. Values are mean ± SD. ^a,b^Values in the same row with different superscripts differ significantly (*P* < 0.05).

### Fecal scores across different treatments

**Figure 1:**
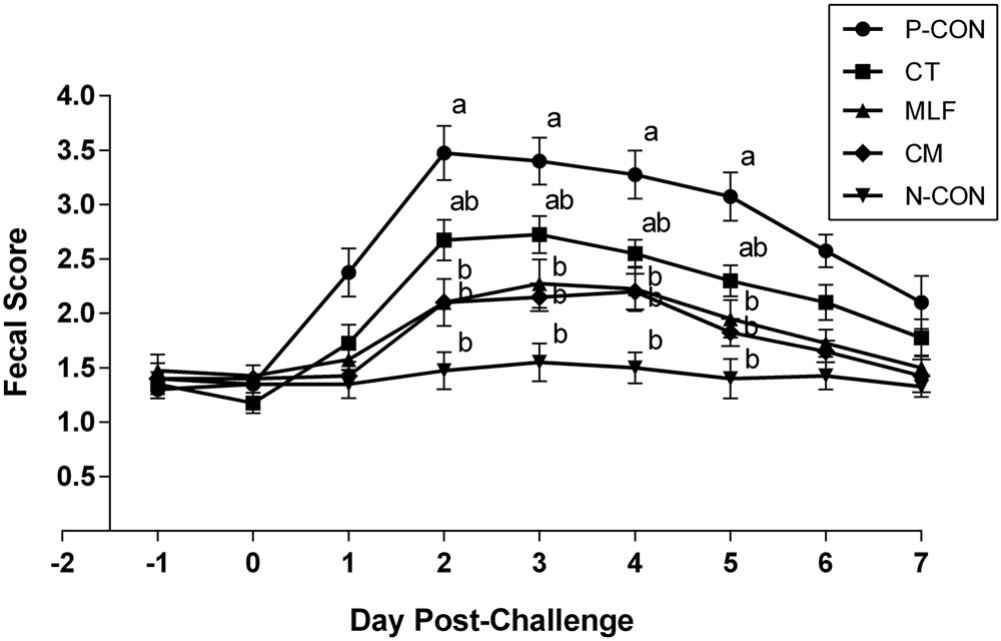
The effect of feeding *Candida tropicalis* and mulberry leaf flavonoids, singly or in combination, on fecal scores of dairy calves challenged with *E. coli* K99. N-CON: fed a basal diet and not challenged with *E.coli* K99; P-CON: fed a basal diet and challenged with *E.coli* K99; CT, MLF, and CM: fed a basal diet supplemented with *C.tropicalis*, mulberry leaf flavonoids, and the combination of the two additives, respectively, and challenged with *E.coli* K99. The X-axis denotes the day post-challenge and the Y-axis the fecal score. Values are mean ± SD. ^a,b^Values in the same column with different superscripts differ significantly (*P* < 0.05).

In order to observe whether dietary supplementation with the two alternatives to antibiotics would have effects on prevention of diarrhea in preweaned calves challenged with *E. coli*, fecal scores were performed after *E. coli* K99 challenge. Calves from the P-CON group suffered from diarrhea on the first day after the *E. coli* K99 challenge, and all the other calves challenged with *E. coli* K99 experienced diarrhea on the second day. However, calves fed *C. tropicalis* (CT), mulberry leaf flavonoids (MLF), or the combination (CM) had lower fecal scores and experienced fewer days with fecal score >2 than that in the P-CON group (Fig. 1). Fecal scores of the MLF and CM groups were significantly lower (*P* < 0.05) when compared with the P-CON group on d 2–5 after the *E. coli* K99 challenge. Dietary supplementation with *C. tropicalis* and mulberry leaf flavonoids, singly or in combination, improved fecal scores and reduced the number of days with mild or watery diarrhea.

### Sequencing depth and alpha diversity

Illumina MiSeq sequencing analysis of the 24 jejunum digesta samples of preweaned calves generated a total of 1017,001 trimmed reads with an average of 42,622 ± 12,268 reads per sample after data filtering, quality control, and removal of primers, chimeras, and low-confidence singletons. For further analyses, all reads were classified into 512 operational taxonomic units (OTUs) based on ≥ 97% nucleotide sequence identity between reads.

**Figure 2:**
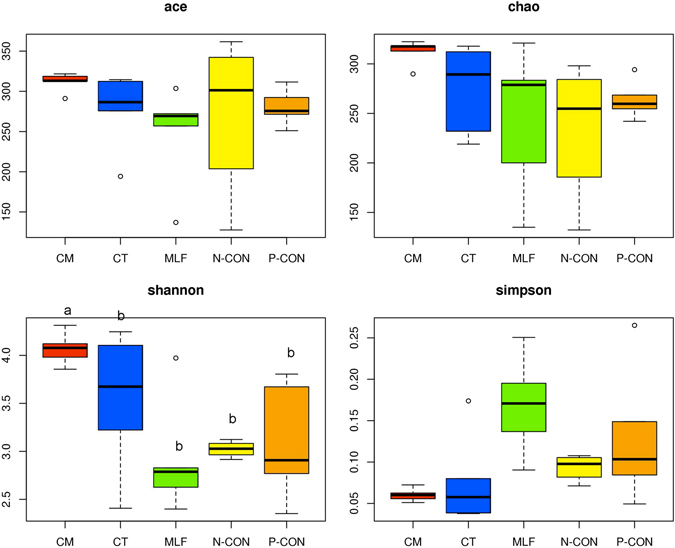
Community richness estimates (ACE and Chao1) and diversity indices (Shannon and Simpson) for different treatments. ^a,b^Boxes with different superscripts differ significantly (*P* < 0.05).

We assessed whether our sequencing depth provided sufficient diversity coverage to accurately describe the bacterial composition of each group by generating sample-based rarefaction curves for each group (Supplementary Fig. S1). The results indicated a sufficient sequencing depth for the samples from different groups. The OTU numbers of N-CON, P-CON, CT, MLF, and CM were 154, 208, 217, 189, and 289, respectively. The CM group had significantly higher OTU numbers compared with other groups (*P* < 0.05). The community diversity (Shannon index) differed significantly (*P* < 0.05) between the CM group and other groups in preweaned calves (Fig. 2).

### Gut bacterial composition across different treatments

**Figure 3:**
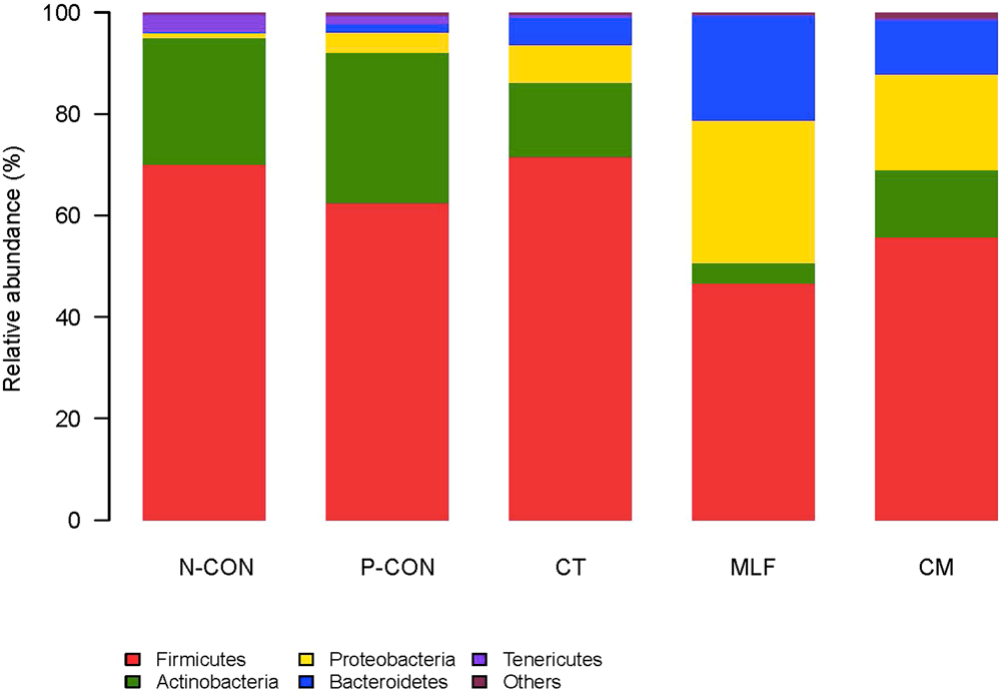
Phylum level composition. Color-coded bar plot showing the relative abundances of the five most abundant phyla across different groups.

At the phylum level, 15 phyla were identified in the samples from the preweaned calf guts, and these were dominated by *Firmicutes*, *Actinobacteria*, *Proteobacteria*, and *Bacteroidetes*, regardless of treatment group (Fig. 3 and Table 2). However, the relative abundance of these predominant phyla varied considerably among the different calf groups. The *Firmicutes* dominated in all treatment groups (Table 2). The phylum *Actinobacteria* was abundant in the samples taken from the N-CON and P-CON groups when compared with the MLF, CT and CM groups, and this phylum was significantly lower (*P* < 0.05) in the MLF group than in the N-CON and P-CON groups. The phyla *Bacteroidetes* and *Proteobacteria* were abundant in samples taken from the MLF, CT, and CM groups when compared with the N-CON and P-CON groups, and the two phyla were more abundant in the MLF and CM groups (*P* < 0.05) than in the other groups. Among other minor phyla, the TM7 and *Verrucomicrobia* were more prominent in the MLF, CT and CM groups, while the *Tenericutes* and *Synergistetes* were more prominent in the N-CON and P-CON groups.

**Table 2:** Phylum-level composition of the jejunum digesta among different groups.

Phylum	Relative abundance (%)					*P*
	N-CON	P-CON	CT	MLF	CM	
*Acidobacteria*	0.00 ± 0.00^b^	0.03 ± 0.02^b^	0.02 ± 0.02^b^	0.04 ± 0.03^b^	0.13 ± 0.08^a^	0.0103
*Actinobacteria*	24.86 ± 2.75^a^	29.59 ± 16.18^a^	14.53 ± 12.38^ab^	4.07 ± 2.30^b^	13.23 ± 10.67^ab^	0.0112
*Bacteroidetes*	0.36 ± 0.23^c^	1.65 ± 1.51^c^	5.37 ± 4.47^bc^	20.62 ± 17.28^a^	10.82 ± 2.87^ab^	0.0022
*Chloroflexi*	0.02 ± 0.00	0.07 ± 0.05	0.03 ± 0.02	0.06 ± 0.04	0.15 ± 0.12	0.1822
*Firmicutes*	70.07 ± 7.78	62.54 ± 15.51	71.59 ± 9.20	46.59 ± 28.78	55.70 ± 6.42	0.1798
*Proteobacteria*	0.99 ± 0.30^c^	3.97 ± 3.29^bc^	7.43 ± 4.54^b^	28.10 ± 14.77^a^	18.85 ± 2.62^a^	<0.0001
*Synergistetes*	0.24 ± 0.18	0.19 ± 0.13	0.05 ± 0.02	0.06 ± 0.04	0.01 ± 0.00	0.4400
*Tenericutes*	3.36 ± 2.18	1.69 ± 0.84	0.62 ± 0.57	0.16 ± 0.16	0.28 ± 0.11	0.2553
TM7	0.05 ± 0.02	0.07 ± 0.03	0.21 ± 0.14	0.08 ± 0.06	0.28 ± 0.19	0.1281
*Verrucomicrobia*	0.02 ± 0.02^b^	0.05 ± 0.05^b^	0.04 ± 0.03^b^	0.07 ± 0.06^b^	0.22 ± 0.09^a^	0.0468

Values are mean ± SD. ^a,b^Values in the same row with different superscripts differ significantly (*P* < 0.05).

**Figure 4:**
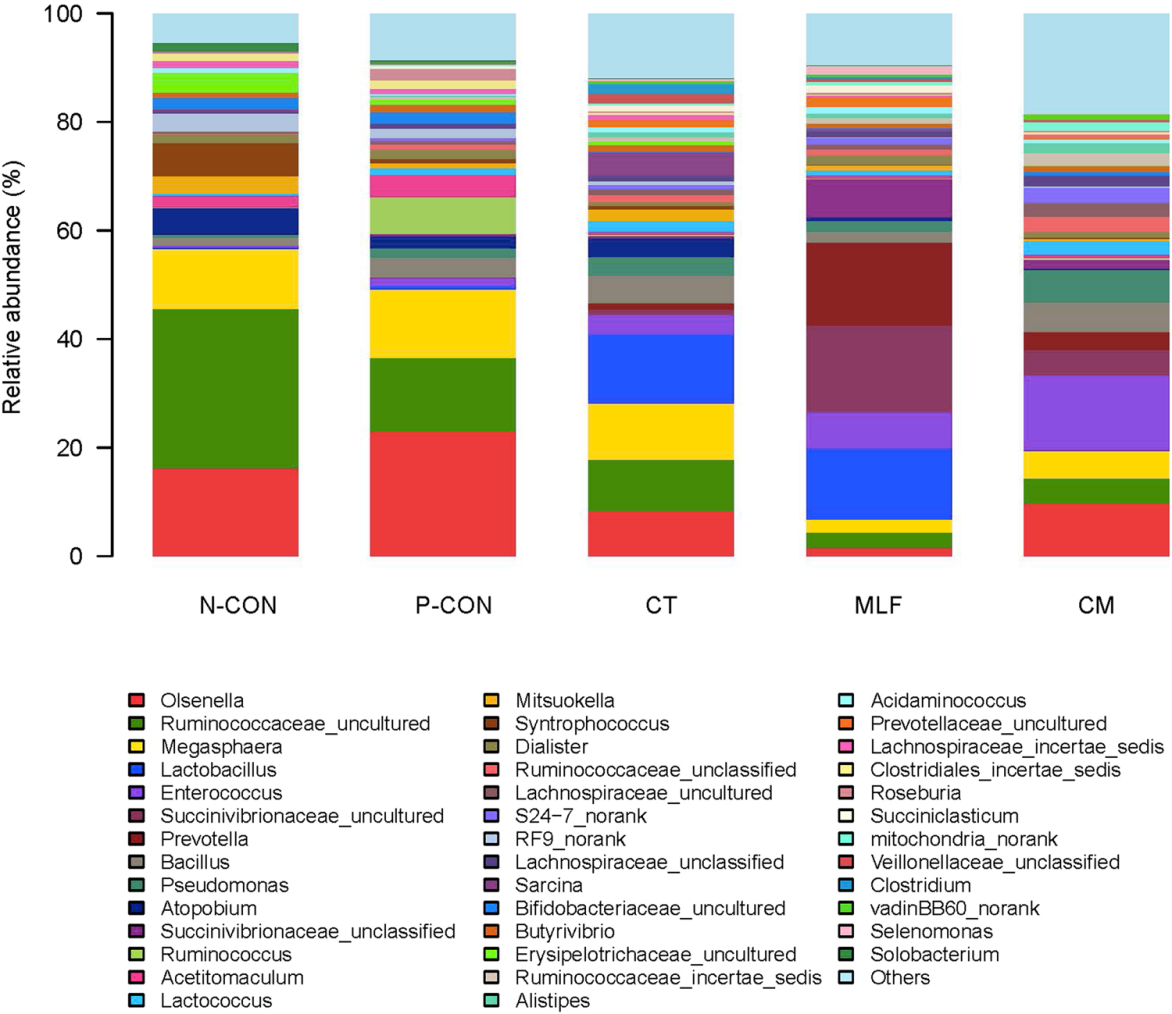
Genus level composition. Color-coded bar plot showing the relative abundances of different genera across different groups.

At the genus level, 185 genera belonging to the 15 phyla were detected in the samples. In total, 25 most abundant shared genera with a relative abundance ≥ 0.1% (Table 3) were present in all samples across different groups, but their relative abundance levels were markedly different among the different treatment groups (Fig. 4 and Table 3). The *Prevotella*, *Enterococcus*, and *Lactobacillus* made up the main bacterial species in MLF group, with *Prevotella* and *Lactobacillus* having the highest relative abundances in this group. The *Enterococcus* and *Pseudomonas* were the two most abundant genera in the samples from the CM group.

**Table 3:** Shared genera with a relative abundance ≥ 0.1% in all samples among different groups.

Phylum	Genus	Relative abundance (%)					*P*
		N-CON	P-CON	CT	MLF	CM	
*Actinobacteria*	*Arthrobacter*	0.11 ± 0.05	0.30 ± 0.16	0.41 ± 0.33	0.23 ± 0.25	0.55 ± 0.07	0.0945
	*Atopobium*	4.83 ± 2.31	2.27 ± 1.15	3.45 ± 2.88	0.79 ± 0.56	0.33 ± 0.13	0.0072
	*Bifidobacterium*	0.40 ± 0.19	0.76 ± 0.74	0.51 ± 0.40	0.54 ± 0.32	0.45 ± 0.45	0.9593
	*Corynebacterium*	0.32 ± 0.28	0.36 ± 0.12	0.34 ± 0.16	0.17 ± 0.23	0.49 ± 0.25	0.3389
	*Olsenella*	16.24 ± 6.21^a^	22.93 ± 14.68^a^	10.50 ± 9.04^ab^	2.02 ± 1.93^b^	9.72 ± 8.05^ab^	0.0341
*Bacteroidetes*	*Alistipes*	0.12 ± 0.07^b^	0.29 ± 0.27^b^	1.11 ± 0.83^ab^	1.03 ± 0.78^ab^	1.89 ± 0.89^a^	0.0640
	*Prevotella*	0.05 ± 0.03^b^	0.06 ± 0.05^b^	2.06 ± 2.02^b^	15.22 ± 7.31^a^	3.23 ± 2.87^b^	0.0039
	*Myroides*	0.08 ± 0.04	0.23 ± 0.13	0.20 ± 0.18	0.20 ± 0.19	0.34 ± 0.16	0.4611
*Firmicutes*	*Acetitomaculum*	2.05 ± 1.94	4.07 ± 3.69	0.71 ± 0.43	0.56 ± 0.20	0.65 ± 0.10	0.0682
	*Acidaminococcus*	0.70 ± 0.47	0.32 ± 0.21	0.99 ± 0.68	1.60 ± 0.82	1.18 ± 0.92	0.8772
	*Bacillus*	1.43 ± 0.39	3.58 ± 2.61	5.03 ± 3.63	2.02 ± 1.51	5.55 ± 2.72	0.2204
	*Butyrivibrio*	0.93 ± 0.52	1.54 ± 1.32	1.17 ± 1.19	0.74 ± 0.66	1.05 ± 1.17	0.8966
	*Dialister*	2.14 ± 1.76	1.78 ± 0.88	0.62 ± 0.59	2.10 ± 1.25	1.02 ± 0.90	0.6678
	*Enterococcus*	0.35 ± 0.14^b^	1.42 ± 1.21^b^	3.63 ± 2.12^b^	8.28 ± 6.47^b^	13.83 ± 6.37^a^	0.0209
	*Howardella*	0.54 ± 0.27^a^	0.34 ± 0.28^ab^	0.16 ± 0.09^b^	0.22 ± 0.26^ab^	0.07 ± 0.05^b^	0.0501
	*Lactobacillus*	0.30 ± 0.25^b^	0.73 ± 0.47^b^	12.70 ± 6.43^a^	13.14 ± 7.26^a^	0.16 ± 0.07^b^	0.0121
	*Lactococcus*	0.45 ± 0.13^b^	1.21 ± 0.83^ab^	2.06 ± 1.54^ab^	0.94 ± 0.40^ab^	2.52 ± 0.92^a^	0.0931
	*Megasphaera*	11.08 ± 9.95	12.58 ± 9.10	10.32 ± 8.07	2.48 ± 2.36	5.04 ± 3.57	0.2282
	*Mitsuokella*	3.23 ± 2.34	0.93 ± 0.72	2.08 ± 1.07	1.14 ± 0.68	0.41 ± 0.39	0.3383
	*Pseudoramibacter*	0.43 ± 0.23^ab^	0.85 ± 0.76^a^	0.49 ± 0.17^ab^	0.11 ± 0.05^b^	0.16 ± 0.14^b^	0.0451
	*Roseburia*	0.26 ± 0.18	2.12 ± 1.79	0.33 ± 0.29	0.33 ± 0.28	0.28 ± 0.15	0.7039
	*Ruminococcus*	0.27 ± 0.25	0.11 ± 0.03	0.25 ± 0.14	0.17 ± 0.12	0.41 ± 0.16	0.2007
	*Syntrophococcus*	6.03 ± 5.03^a^	0.78 ± 0.17^b^	0.68 ± 0.28^b^	0.21 ± 0.11^b^	0.27 ± 0.21^b^	0.0002
*Proteobacteria*	*Pseudomonas*	0.64 ± 0.16^b^	1.77 ± 1.32^b^	3.42 ± 0.36^a^	2.60 ± 2.45^ab^	5.94 ± 1.28^a^	0.0006
	*Psychrobacter*	0.11 ± 0.09	0.17 ± 0.06	0.26 ± 0.22	0.10 ± 0.08	0.42 ± 0.27	0.2002

Values are mean ± SD. ^a,b^Values in the same row with different superscripts differ significantly (*P* < 0.05).

### Quantification of total bacteria, *E.coli* K99, and the four selected bacterial species

**Figure 5:**
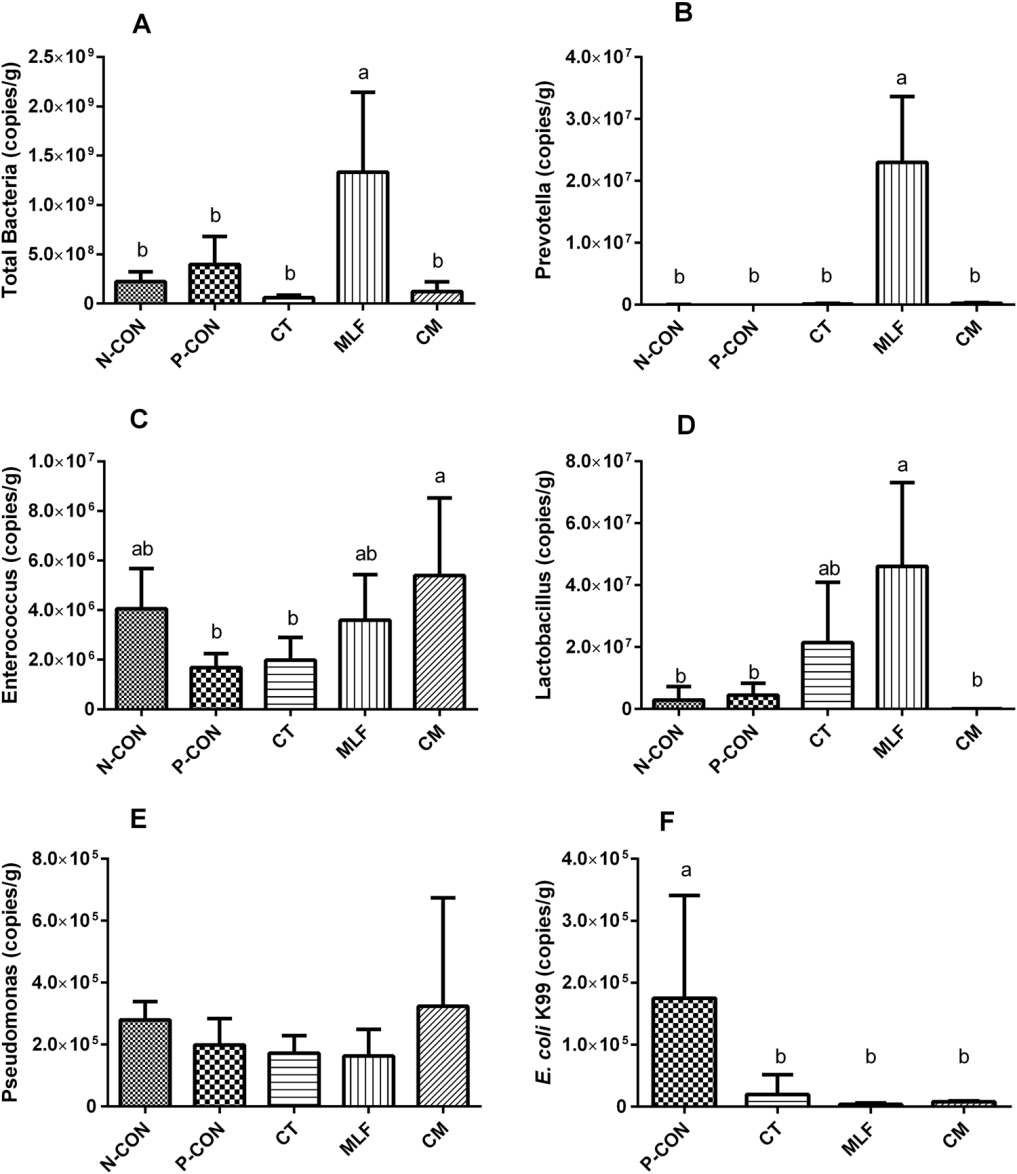
The copy numbers of selected bacterial species and of *E.coli* K99 in the jejunum digesta. A to F represent the copy numbers per gram of total bacteria, *Prevotella*, *Enterococcus*, *Lactobacillus*, *Pseudomonas*, and *E.coli* K99, respectively. In figure F, no *E. coli* K99 was detected in N-CON group. Values are mean ± SD. ^a,b^Bars with different superscripts differ significantly (*P* < 0.05).

We used absolute quantitative real-time PCR to investigate the quantification of total bacteria, *E.coli* K99, and four selected bacterial species that showed statistically significant in sequencing results (Fig. 5). The copy numbers of total bacteria and of the genus *Prevotella* were significantly higher in MLF group than in other groups. The genus *Enterococcus* had significantly higher copy numbers in CM group than in P-CON and CT groups. The genus *Lactobacillus* had significantly higher copy numbers in MLF group than in N-CON, P-CON, and CM groups. The copy numbers of *Pseudomonas* were no significant difference across different treatments. The relative abundances of the four bacterial species calculated by quantitative real-time PCR (Supplementary Table S1) had the similar statistical differences with that calculated by Illumina MiSeq sequencing. No *E. coli* K99 was detected in the digesta samples taken from the N-CON group. The copy numbers of the *E.coli* K99 were significantly higher in P-CON group than that in CT, MLF, and CM groups (Fig. 5).

## Discussion

A role in disease has been reported for the intestinal microbiota, whereby gut microbes function as a key interface between host and environment and some bacteria protect the host from pathogens that cause infectious diarrhea^39^. The use of antibiotics has already been reported to affect intestinal microbiota profiles in humans^40^ and swine^41^. Evidence is also emerging that antibiotic usage in animal production may contribute to the antibiotic resistance of human pathogens^42^. Alternatives to antibiotics for the prevention and treatment of disease in young calves are continuously being evaluated and are urgently required in order to minimize the need for antibiotics^43^. This need prompted the present analysis of the effects of two potential antibiotic alternatives–*C. tropicalis* and mulberry leaf flavonoids, supplied singly or in combination–on the intestinal bacterial community composition in preweaned calves challenged with *E. coli* K99.

In the present study, the MLF and CM groups had significantly higher ADG and feed efficiency, and significantly lower fecal scores compared with the P-CON group after *E. coli* K99 challenge. Diet supplementation with *C. tropicalis* and mulberry leaf flavonoids, singly or in combination, significantly increased the relative abundance of *Lactobacillus* and *Enterococcus*, which belong to lactic acid producing bacteria (LAB). Many studies indicated that diet supplemented with LAB could improve weight gain and feed efficiency, and reduced diarrhea incidence^44–46^. Furthermore, the structure of flavonoids is similar to estradiol, which can regulate the secretion of growth hormone by the hypothalamus-pituitary hormone axis^47^. Growth hormone directly accelerates protein synthesis or stimulates insulin-like growth factors 1 to promote muscle tissue growth and body weight gain^48^.

Dietary supplementation with *C. tropicalis* and mulberry leaf flavonoids, singly or in combination, improved fecal scores and reduced the number of days with mild or watery diarrhea, suggesting protective effects of both supplements in calves with a high risk of morbidity. Previous studies showed that a yeast culture supplement decreased the risk of diarrhea due to *E. coli*, because the *E. coli* adhered to the oligosaccharides present in the yeast cell walls rather than attaching to and invading the intestinal cells^49, 50^. This might be the reason that the copy number of *E. coli* K99 in CT group was significantly lower than that in the P-CON group. A considerable number of studies have demonstrated that flavonoids isolated from the leaves of many different plants show good antimicrobial activity against *E. coli* ^26, 31, 33, 34^. The antimicrobial activity of flavonoids is apparently due to their ability to penetrate biological membranes^35^. The antimicrobial activity of flavonoids might be one of the reasons that the MLF group had significantly lower copy number of *E. coli* K99 compared with the P-CON group. As mentioned above, feeding *C. tropicalis*, mulberry leaf flavonoids, or their combination increased the relative abundance of LAB in the calf gut. The LAB can adhere to the intestinal tract and produce lactate and acetate, which reduces the attachment and colonization of *E. coli* on the surface of intestinal epithelial cells^44, 46^. The increasing abundance of LAB in the calf gut might be another reason that the CT, MLF, and CM groups had significantly lower copy number of *E. coli* K99 compared with the P-CON group. The observed increase in LAB abundance would be expected to improve gut health and may explain the benefits seen in fecal scores and diarrhea observed in this study when calves were fed either of the two supplements or their combination.

The community diversity index (Fig. 2) and OTU number significantly increased in the CM group compared with all other groups, suggesting that the combination of *C. tropicalis* and mulberry leaf flavonoid supplements increased the intestinal community diversity in preweaned calves, whereas each supplement on its own did not have this effect. As mentioned above, flavonoids isolated from the leaves of many plants exhibit antimicrobial activity^29–34^, which means flavonoids might decrease the community diversity. However, in the current study, dietary supplementation with the combination of *C. tropicalis* and mulberry leaf flavonoid increased the intestinal community diversity. The reason might be the synergistic effect of *C. tropicalis* and mulberry leaf flavonoid. The action mechanism needs to be further studied in future.

The OTU number and community diversity of the gastrointestinal tracts of the calves in the present study were lower than previously reported in some studies. One reason might be that the previous studies examined weaned calves or adult cows, and that the species richness and diversity in the gut increases with age^51–53^. The gradual increase in consumption of large amounts of different solid feeds might be one reason for an age-dependent increase in bacterial diversity^54, 55^. Another reason might be that the digesta samples were collected from different gastrointestinal tract regions in different studies. Malmuthuge *et al*. reported that the highest number of OTUs and the greatest bacterial community diversity indices were observed in the rumen, followed by the large intestine (cecum and colon), and then the small intestine (jejunum and ileum)^56^. The rumen and large intestine are regarded as fermentation tanks for microbial fermentation of indigestible dietary substrates, and the retention time of digesta is longer in the rumen and large intestine than in the small intestine, which would facilitate the growth of a more complex bacterial community^56^.

The dominant phyla found in all groups were *Firmicutes*, *Actinobacteria*, *Proteobacteria*, and *Bacteroidetes*. The groups shared 113 genera, and the 25 most abundant shared genera, with a relative abundance ≥ 0.1%, were present in all samples across the different groups. These dominant phyla and shared genera represented the core microbiome of the calves of this age, irrespective of the treatments. However, the relative abundance of the phyla and genera from this shared community varied considerably among the groups.

In the present study, *Firmicutes* showed the highest overall relative abundance and dominated in all the treatment groups. Our findings are consistent with the studies of Oikonomou *et al*. and Malmuthuge *et al*., who detected a significantly higher relative abundance of *Firmicutes* in the gut digesta of preweaned Holstein calves^53, 56^. Malmuthuge *et al*. implied that *Firmicutes* tended to more readily colonize the small intestinal digesta of preweaned calves, while *Bacteroidetes* tended to more readily colonize the rumen and large intestinal contents^56^. The regional variations in the gastrointestinal tract of preweaned calves revealed differences in the dominant bacterial community^56^.

The LABs, such as *Lactobacillus* and *Enterococcus*, have known beneficial effects on feed efficiency and animal health^46^, and both *Lactobacillus* and *Enterococcus* have been used as direct-fed microbials for a long time. Studies have reported that direct feeding of calves or dairy cows with microbials consisting of *Lactobacillus* and *Enterococcus* prevented declines in ruminal pH and decreased the risk of metabolic acidosis. These effects occurred due to facilitation of the growth of ruminal microorganisms adapted to the presence of lactic acid and by stimulation of the utilization of lactic acid by lactic acid utilizing bacteria^46, 57, 58^. Calves fed *Lactobacillus* and *Enterococcus* also showed an improved abundance of ruminal cellulolytic bacteria, such as *Butyrivibrio fibriosolvens* and *Eubacterium ruminantium* ^58^. In our study, the relative abundance of *Lactobacillus* increased in the CT and MLF groups, and the relative abundance of *Enterococcus* increased in the CT, MLF, and CM groups.

The phylum *Bacteroidetes* was significantly more abundant in the MLF and CM groups, and especially in the MLF group, when compared to the N-CON and P-CON groups. *Bacteroidetes* was composed mainly of the genera *Alistipes*, *Myroides*, and *Prevotella* in all groups, and the genus *Prevotella* was significantly more abundant in the MLF group, accounting for up to 15.22% of the total reads. The *Prevotella* genus contains several ruminal species that are capable of utilizing starches, other non-cellulosic polysaccharides, and simple sugars as energy sources^59^, and this genus was more abundant in the CT, MLF, and CM groups than in the N-CON and P-CON groups.

Previous studies have reported that the *Bacteroidetes* found in the rumens of preweaned calves fed whole milk or milk replacer contain more *Bacteroides* than *Prevotella* ^56, 60^, whereas the rumens of adult cattle contain almost exclusively *Prevotella* ^51^. A recent study showed that the relative abundance of *Bacteroides* in the feces of adult cattle was negatively associated with a high fiber diet^54^. The diet of preweaned calves, which mainly includes whole milk or milk replacer, is rich in protein, fat, and sugar, whereas the adult cattle’s diet is composed mainly of plant fiber. In the present study, the small intestinal digesta of the 9-week-old preweaned calves contained more *Prevotella* than *Bacteroides* and the diet of the calves consisted of milk replacer, calf starter, and hay, with the calf starter and hay dominating, suggesting that, during calf development, increased fiber ingestion and decreased milk consumption decreases the relative abundance of *Bacteroides* ^55^.

Other phyla, such as the *Actinobacteria* and *Proteobacteria*, were also found in high proportion in all treatment groups. The relative abundance of *Actinobacteria* decreased and *Proteobacteria* increased in the MLF, CT, and CM groups, and especially in the MLF group, when compared with the N-CON and P-CON groups, indicating that the dietary supplements helped to encourage the growth of *Proteobacteria* and to inhibit the growth of *Actinobacteria* in the preweaned calves.

## Conclusions

The results presented here provide new information regarding the effects of dietary supplementation with two alternatives to antibiotics (*C. tropicalis* and mulberry leaf flavonoids), and their combination on the intestinal microbiota in preweaned calves challenged with *E. coli* K99. The MLF and CM groups had significantly higher ADG and feed efficiency compared with the P-CON group after *E. coli* K99 challenge. Dietary supplementation with the two alternatives to antibiotics, singly or in combination, improved fecal scores and reduced days with mild or watery diarrhea. Dietary supplementation with the combination of *C. tropicalis* and mulberry leaf flavonoids significantly increased the number of OTUs and the community diversity. Dietary supplementation with the two alternatives to antibiotics, singly or in combination, increased the relative abundance, at the phylum level, of *Bacteroidetes* and *Proteobacteria* and decreased the relative abundance of *Actinobacteria*, while at the genus level, this supplementation increased the relative abundance of *Prevotella*, *Lactobacillus*, and *Enterococcus*. Quantitative real-time PCR revealed that dietary supplementation with mulberry leaf flavonoids significantly increased the copy numbers of total bacteria and of the genera *Prevotella* and *Lactobacillus* in jejunum digesta. The CT, MLF, and CM groups had significantly lower copy numbers of *E.coli* K99 compared with the P-CON group. Our results establish a strong foundation for evaluating the potential of *C. tropicalis* and mulberry leaf flavonoids as feed additives for the reduction of diarrhea and improvement of intestinal health in preweaned calves challenged with *E. coli* K99.

## Materials and Methods

### Animal experiment and sample collection

The experiments were approved by the Animal Ethics Committee of the Chinese Academy of Agricultural Sciences, Beijing, China. All methods were performed in accordance with the relevant standard operating procedures approved by the above mentioned ethics committee.

Sixty newborn Holstein bull calves with body weight 40 ± 2.0 kg were purchased from a commercial dairy farm, fed colostrum within 2 h after birth and for the first 3 d of life. The calves were then housed individually in 1.6 m × 3.6 m pens with wood shavings for bedding at the experimental farm of Chinese Academy of Agricultural Sciences and fed with commercial pasteurized whole milk twice a day until d 21. Over the following week, the pasteurized whole milk was gradually replaced with milk replacer (1:7 w/v; Table 4), which was then fed twice daily at a total amount corresponding to 10% of the calves’ body weight until d 64. All calves had *ad libitum* access to calf starter (Table 4) and hay from d 4 to 64. Clean fresh water was offered free choice daily throughout the study.

**Table 4:** Ingredients and chemical composition of starter and milk replacer.

Item	Starter (%)	Milk replacer (%)
Ingredient		
Corn	20.00	
Extruded corn	22.90	
Soybean meal	20.00	
Extruded soybean	18.00	
Dried whey	5.00	
Wheat bran	10.00	
Calcium hydrogen phosphate	0.80	
Limestone	1.80	
Salt	0.50	
Premix^1^	1.00	
Chemical composition		
DM^2^	85.36	95.36
OM	92.21	94.85
CP	19.08	24.27
Ether extract	2.21	12.85
NDF	18.59	4.02
ADF	10.65	2.11
Calcium	1.09	1.07
Phosphorous	0.47	0.48
Gross energy, MJ/kg	15.45	19.86

^1^Premix was manufactured by the Precision Animal Nutrition Research Centre, Beijing, China. Premix provided per kilogram of concentrate: vitamin A, 15,000 IU; vitamin D, 5,000 IU; vitamin E, 50 mg; Fe, 90 mg; Cu, 12.5 mg; Mn, 30 mg; Zn, 90 mg; Se, 0.3 mg; I, 1.0 mg. ^2^DM = dry matter; OM = organic matter; CP = crude protein; NDF = neutral detergent fiber; ADF = acid detergent fiber.

At 28 d of age, 60 calves were randomly assigned to 5 treatments with 12 calves each based on BW and date of birth. The treatments were as follows: 1) negative control (N-CON) treatment: fed a basal diet and not challenged with *E. coli* K99; 2) positive control (P-CON): fed a basal diet and orally challenged with *E. coli* K99 (30 mL; 1 × 10^9^ CFU/mL); 3) *C. tropicalis* treatment (CT): fed a basal diet supplemented daily with *C. tropicalis* (5.0 × 10^9^ CFU/g; 1 g/calf) and then orally challenged with *E. coli* K99 (30 mL; 1.0 × 10^9^ CFU/mL); 4) mulberry leaf flavonoid treatment (MLF): fed a basal diet supplemented daily with mulberry leaf flavonoids (5.0%, w/w; 3 g/calf) and then orally challenged with *E. coli* K99 (30 mL; 1.0 × 10^9^ CFU/mL); and 5) combined *C. tropicalis* and mulberry leaf flavonoid treatment (CM): fed a basal diet supplemented daily with both *C. tropicalis* (5.0 × 10^9^ CFU/g; 1 g/calf) and mulberry leaf flavonoids (5.0%, w/w; 3 g/calf) and then orally challenged with *E. coli* K99 (30 mL; 1.0 × 10^9^ CFU/mL). The basal diet (Table 4) was free of antibiotics or other additives. Experimental treatments were applied from d 28 to 64, with the oral challenges with *E. coli* K99 carried out on d 57.

**Figure 6:**
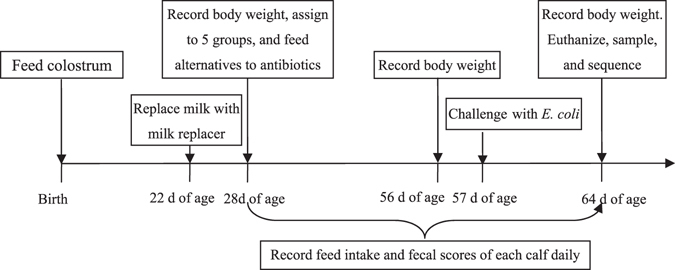
The flow scheme of this study. Calves were fed colostrum within 2 h after birth. At 22 d of age, whole milk was gradually replaced with milk replacer, which was then fed twice daily until d 64. At d 28, 56, and 64 of age, body weight of each calf was recorded. From d 28 to 64 of age, feed intake and fecal scores were recorded. At 28 d of age, calves were randomly assigned to 5 treatments and treated with alternatives to antibiotics. At 57 d of age, *E. coli* K99 challenge were carried out. At 64 d of age, calves were euthanized and jejunum digesta samples were collected and sequenced.

The body weight of each calf was recorded at d 28, 56, and 64 of age. Feed intake and fecal scores of each calf was recorded daily. Five calves were selected from each group and euthanized at 64 d of age. Jejunum digesta samples were collected from the middle of the jejunum and at the same site consistently for all the animals. Jejunum digesta samples were snap frozen in liquid nitrogen and kept at −80 °C until further analysis. The flow scheme of this trial was shown in Fig. 6.

### Additives preparation and oral challenge

A commercial CT probiotic (5.0 × 10^9^ CFU/g) was purchased from Beijing Huanong Biological Engineering Co., Ltd., Beijing, China. Commercial mulberry (*Morus alba* Linn.) leaf extracts were purchased from Xi’an Feida Biotechnology Co. Ltd., Xi’an, China. The mulberry leaf extracts were vacuum-dried and contained 5.0% flavonoids (w/w). The CT and MLF were mixed with the milk replacer and administered with the morning feeding.

The *E. coli* K99 strain was obtained from China Veterinary Culture Collection Center. The viability of the culture was ensured by growing it aerobically in Luria Bertani broth for 24 h at 37 °C with shaking (120 rpm). A growth curve was constructed for *E. coli* K99 to determine the appropriate incubation time required to reach the target challenge dosage of approximately 1.0 × 10^9^ CFU/mL. *E. coli* K99 (30 mL, 1.0 × 10^9^ CFU/mL) was mixed with milk replacer and fed to the calves on d 57.

### Fecal consistency scoring

Fecal consistency scoring was performed daily before the morning milk feeding using a 1 to 4 scale, as described by Magalhaes *et al*.^50^. Briefly, fecal consistency was scored as 1 when firm, 2 when soft or of moderate consistency, 3 when runny or mild diarrhea, and 4 when watery and profuse diarrhea. Daily fecal scores were generated for individual calves for statistical analyses. Calves with fecal score >2 were used for analysis of incidence of diarrhea.

### DNA extraction, PCR amplification, and Illumina miseq sequencing

Microbial DNA was extracted from jejunum digesta samples using the OMEGA E.Z.N.A.® digesta DNA Kit (Omega Bio-tek, Norcross, GA, USA), according to the manufacturer’s protocols. The quality and quantity of the DNA were measured using an ND1000 spectrophotometer (NanoDrop Technologies Inc., Wilmington, DE, USA). The V3–V4 regions of the bacterial 16S ribosomal RNA gene were amplified by PCR (95 °C for 3 min, followed by 27 cycles at 95 °C for 30 s, 55 °C for 30 s, and 72 °C for 45 s and a final extension at 72 °C for 10 min) using primers 338 F 5′-barcode-ACTCCTACGGGAGGCAGCAG-3′ and 806 R 5′-barcode-GGACTACHVGGGTWTCTAAT-3′^61^, where barcode is an eight-base sequence unique to each sample. PCR reactions were performed in triplicate 20 μl mixtures containing 4 μl 5×FastPfu Buffer, 2 μl 2.5 mM dNTPs, 0.8 μl of each primer (5 μM), 0.4 μl FastPfu Polymerase, and 10 ng template DNA.

Amplicons were extracted from 2% agarose gels, purified using the AxyPrep DNA Gel Extraction Kit (Axygen Biosciences, Union City, CA, USA) according to the manufacturer’s instructions, and quantified using the QuantiFluor™ -ST system (Promega, Madison, WI, USA). Purified amplicons were pooled in equimolar and paired-end sequenced (2 × 300 bp) on an Illumina MiSeq PE300 platform (Illumina, Inc., San Diego, CA, USA) according to the standard protocols.

### Processing of sequencing data

After raw FASTQ files demultiplexed, sequences were filtered using Trimmomatic^62^ following the criteria: (i) the 300-bp reads were truncated at any site receiving an average quality score of < 20 over a 50-bp sliding window, and truncated reads shorter than 50 bp were discarded; (ii) reads containing any mismatch in the barcode region, two or more nucleotide mismatches in the primer sequence, or ambiguous characters were removed; and (iii) only sequences with an overlap of > 10 bp and < 10% mismatches were assembled and reads that could not be assembled were discarded. The assembled sequences were then trimmed of primers and barcodes. Chimeric sequences were identified and removed using usearch^63^. After quality control, the assembled sequences were assigned to operational taxonomic units (OTUs) at a 97% identity threshold using UPARSE^64^. Alpha diversity index, including ACE, Chao1, Shannon, and Simpson were calculated by normalizing the number of reads in all samples to 7668 sequences using mothur^65^. Rarefaction curves were analyzed with mothur and plotted using R. The taxonomy of each 16S rRNA gene sequence was assigned against the SILVA bacteria alignment database^66^ using RDP classifier^67^ with a confidence threshold of 70%. Sequences were aligned against the PyNAST^68^, and a phylogenetic tree was built using FastTree^69^. The sequencing data obtained in this study were deposited in the NCBI Sequence Read Archive (SRA) under accession numbers SRR5406973 to SRR5406996.

### Real-time PCR

Absolute quantitative real-time PCR analysis was performed to estimate the copy numbers of the total bacteria, *E. coli* K99, and four selected bacterial species in the jejunum digesta using the amplification primers shown in Supplementary Table S2. Briefly, a standard curve was generated for the total bacterial gene, *E.coli* K99, and each individual bacterial strain selected using universal primers. Real-time PCR was performed in a 20 μl reaction mixture containing 10 μl 2 × SG Green qPCR Mix (SinoGene, Beijing, China), 0.5 μl of each primer (10 μM), 0.5 μl 10 ng DNA templates, and 8.5 μl nuclease-free water. Amplification involved one holding cycle at 95 °C for 10 min for initial denaturation and then 40 cycles at 95 °C for 20 s for denaturation followed by annealing at 60 °C for 30 s and extension at 72 °C for 20 s. The copy numbers of the total bacteria, *E.coli* K99, and four bacterial species per gram of jejunum digesta were then calculated. The relative abundances of four bacterial species were calculated by dividing the gene copy number of each bacterial species by the gene copy number of total bacteria.

### Statistical analyses

The body weight and feed intake of calves, the alpha diversity indices, and the quantification of total bacteria, *E. coli* K99, and the four selected bacterial species were analyzed by one-way ANOVA using SAS (version 9.2; SAS Institute Inc., Cary, NC, USA). Statistical differences among the means of the treatments were compared using the Duncan’s Multiple Range Test. The relative abundances of communities do not fit normal distribution and arcsine transformation function were performed before analyses. The transformed data of the abundances of communities at the phylum and genus levels were analyzed by one-way ANOVA using SAS. Daily fecal scores were analyzed by ANOVA using the MIXED procedure of SAS, fitting a Poisson distribution^50^ and square root transformation function with repeated measures for count data. The MIXED procedure model included the fixed effects of treatment and day, interaction between treatment and day, and the random effect of the individual nested within treatment. Treatment differences with *P* < 0.05 were considered statistically significant, and 0.05 ≤ *P* < 0.10 was designated as a tendency.

## Electronic supplementary material

Supplementary Information
