# Supplementary material for: Effects of dietary supplementation with two alternatives to antibiotics on intestinal microbiota of preweaned calves challenged with Escherichia coli K99
Source: Sci Rep. 2017 Jul 14;7:5439. doi: 10.1038/s41598-017-05376-z (PMC5511211; doi:10.1038/s41598-017-05376-z)
Supplement: Supplementary file 1 — Supplementary Information [file 41598_2017_5376_MOESM1_ESM.pdf]

Effects of dietary supplementation with two alternatives to antibiotics on intestinal microbiota of preweaned calves challenged with *Escherichia coli* K99

Yanliang Bi, Chuntao Yang, Qiyu Diao*, and Yan Tu*

Feed Research Institute, Chinese Academy of Agricultural Sciences, Key Laboratory of Feed Biotechnology of the Ministry of Agriculture, 100081 Beijing, China

***** Corresponding authors: Qiyu Diao and Yan Tu

Qiyu Diao: diaoqiyu@caas.cn

Yan Tu: tuyan@caas.cn

Mailing address: Feed Research Institute, Chinese Academy of Agricultural Sciences, NO.12 Zhongguancun South Street, Haidian District, 100081 Beijing, China;

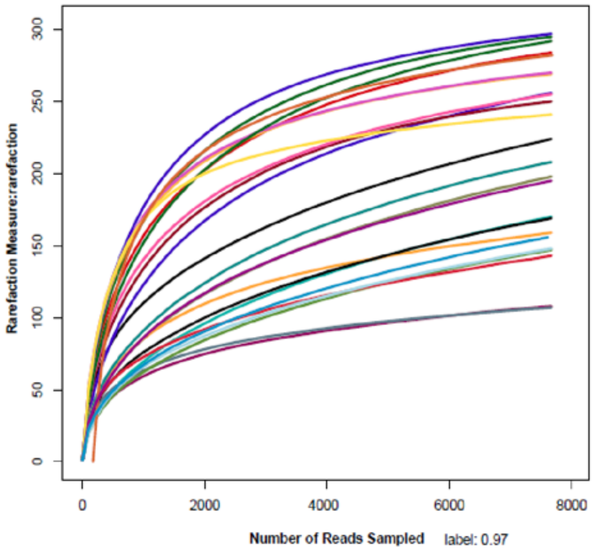

Figure S1. Sample-based rarefaction curves showing the increase in OTU numbers as a function of the number of reads sampled. Each curve represents one individual sample and its corresponding rarefaction calves.

Gene Name	Primer ID	Sequence(5'-3')
total bacteria	Q802	ATGGCTGTCGTCAGCT
	Q803	ACGGGCGGTGTGTAC
Prevotella	Q1954	CACRGTAAACGATGGATGCC
	Q1955	GGTCGGGTTGCAGACC
Enterococcus	Q1956	CCCTTATTGTTAGTTGCCATCATT
	Q1957	ACTCGTTGTACTTCCCATTGT
Lactobacillus	D515	AGCAGTAGGGAATCTTCCA
	D516	CACCGCTACACATGGAG
Pseudomonas	Q1960	AGACACCGTCCAGACTCCTAC
	Q1961	CCAACTTGCTGAACCACCTAC

Table S1. The amplification primers of total bacteria and four selected bacterial species.

Phylum	Relative abundance[1] (%)					P
	N-CON	P-CON	CT	MLF	CM	
Prevotella	0.02 ± 0.01^{b}	0.02 ± 0.01^{b}	0.29 ± 0.23^{b}	1.68 ± 0.75^{a}	0.45 ± 0.24^{b}	0.0056
Enterococcus	0.27 ± 0.19^{b}	2.24 ± 1.14^{b}	4.65 ± 2.96^{b}	1.57 ± 0.65^{b}	5.50 ± 2.39^{a}	0.0251
Lactobacillus	1.19 ± 0.63^{b}	1.57 ± 0.84^{b}	32.14 ± 14.60^{a}	27.97 ± 12.30^{a}	0.15 ± 0.12^{b}	0.0052
Pseudomonas	0.16 ± 0.08^{ab}	0.06 ± 0.02^{b}	0.44 ± 0.23^{a}	0.07 ± 0.02^{b}	0.39 ± 0.23^{a}	0.0218

Table S2. Quantitative real-time PCR results for total bacteria and four selected bacterial species of the jejunum digesta among different groups. [1]The relative abundances of the four selected bacterial species are expressed as percentages, calculated by dividing the gene copy number of each genus by the gene copy number of total bacteria. Values are mean ± SD. [a,b]Values in the same row with different superscripts differ significantly (P<0.05).
